# Environmental toxins and the impact of other endocrine disrupting chemicals in women's reproductive health

**DOI:** 10.5935/1518-0557.20190016

**Published:** 2019

**Authors:** Mauri José Piazza, Almir Antônio Urbanetz

**Affiliations:** 1 Tocogynecology Department, Universidade Federal do Paraná – UFPR – Curitiba (PR), Brazil

**Keywords:** human reproduction, environmental toxicants, environmental pollution, endocrine disrupting chemicals, reproductive health

## Abstract

This review aimed to look into agents and mechanisms characterized as endocrine disrupting chemicals (EDCs). These agents are known to cause several harmful effects to the reproductive system of women and wildlife. There is a wide range of chemicals, developed for commercial use mainly in agriculture, which may cause endocrine disruption. Numerous studies show evidence of environmental contamination. However, no one is being held liable for the damages. The most important potentially harmful agents are identified and described, along with the different effects they have on the female genital area. Brazil is a large consumer of pesticides and others chemicals that may interfere with a normal women's life. We analyzed and described the mode of action and the impacts of different EDCs (bisphenols, phthalates, atrazine, polychlorinated and polybrominated biphenyls, DDT-dichlorodiphenyltrichloroethane; DDE-dichlorodiphenyldichloroethylene; DDD-dichlorodiphenyldichloroethane; and DES-diethylstilbestrol) on the genital area, ovarian steroidogenesis, polycystic ovary syndrome, endometriosis, the structure of the uterus and the vagina, and on the formation of leiomyomas.

## INTRODUCTION

A growing number of scientific evidence has been collected over the past few years suggesting that human reproductive capacity has been affected by a wide range of recurrent substances present in a wide array of everyday products. Several indicators are showing increased incidence of cardiovascular disorders, obesity, hormone-dependent cancers, and chronic diseases, not to mention early puberty development, pregnancy length disorders, and other reproductive health abnormalities.

Among the acting agents are substances such as bisphenol A (BPA) and its byproducts bisphenol B, tetrabromobisphenol A, and bisphenols F and S. All of these and more have been defined as endocrine disrupting chemicals (EDCs). Endocrine disruptors are chemicals that may interfere and cause adverse effects on the endocrine system at any life-stage on account of their resemblance with endogenous steroid hormones. Birnbaum (2013) showed that the global production of these chemicals increased 23.5 fold between 1947 and 2007. In 2012 alone, the US produced 9.5 trillion pounds - 2.09 trillion kilograms - of these chemicals embedded in products such as pesticides, plastics, chemical drugs, and even personal hygiene products.

Deserving more attention are DDT (dichlorodiphenyltrichloroethane), DDE (dichlorodiphenyldichloroethylene), DDD (dichlorodiphenyldichloroethane) and their byproducts such as atrazine and 2,4-dichlorophenoxyacetic acid found in toys, and others containing lead and cadmium, materials used in the production of plastic bottles containing BPA, phthalates, and several other substances employed in the textile and apparel industries (Gore *et al*., 2015). Numerous studies examined the effects of EDCs and their adverse effects against different areas of the female reproductive system.

## Historical Biochemical Features

BPA or 2,2-bis(4-hydroxyphenyl) propane was first synthesized by Dianin, in 1891, and its estrogenic properties were found by Dodds & Lawson (1936). DES (diethylstilbestrol) was also found to have a powerful estrogenic effect (Dodds *et al.*, 1938). Later, in 1950, it was observed that BPA could be polymerized for the manufacturing of plastics given its lightweight, moldability, and impact resistance. Diethylstilbestrol was defined as the first “endocrine disrupting chemical”, since abnormalities such as later development of vaginal adenosis, clear cell adenocarcinoma of the vagina, and/or uterine anomalies, were found in the exposed female offspring of pregnant women treated to prevent miscarriage.

## Endocrine Disruptors

Examples of endocrine disruptors:

### 1. Bisphenols

Bisphenol A (BPA) was the first to be synthesized, but evidences gathered in 1936 showed a low estrogen effect with affinity for the nuclear estrogen receptor. Its effects depend on dosage, targeted tissue, and tissue development on the site where it acts. The occurrence of estrogenic or anti-estrogenic effects depends on the tissue targeted and on their impact on receptors (Rochester *et al.*, 2015). Global production of BPA has steadily grown in recent years on account of its multiple applications in the plastic and manufacturing industries, in food packaging and toys, causing a constant and permanent poisoning of food, water, and the environment. In 1950, it was found that bisphosphonates could be polymerized and, since then, they have been used to make polycarbonate plastics. These plastics have convenient features such as lightweight, moldability, and impact and heat resistance, and are not susceptible to changes over time. About 20% of these plastics are used as a component of epoxy resin, serving as internal coating for plastic containers and bottles. Therefore, it is a liquid and food contaminant present in abnormal levels in human serum analysis according to the literature. BPA is rapidly metabolized to inactive forms with a mean life cycle of approximately 4-5 hours in adults, while in fetuses and children the metabolic rate is relatively low (Gerona *et al*., 2013; Sartain & Hunt, 2016). BPA can easily accumulate in adipose tissue for having lipophilic properties. Measurements of human serum have determined varied and controversial toxicity rates. Currently, the United States Environmental Protection Agency has established a safe level of 50µg/kg/day and the European Food Safety Authority has established a tolerable daily intake below 4µg/kg/day. The list of products with bisphenols available in the market has grown steadily, with the most common being Bisphenol S,F,B, and AF.

### 2. Phthalates

Phthalates and their esters consist of a large group of chemical compounds frequently used in the plastic, coating, cosmetic, and toy industries, including the manufacturing of medical equipment such as syringes and blood bags. Phthalates are byproducts of phthalic acid and are used in the plastics industry for their excellent moldability. There are no regulations restricting the use of phthalates in the United States or in Brazil, but the European Community has banned phthalates. In the roster of phthalates, three esters are considered endocrine disruptors with estrogenic effects: DHEP (diethyl-hexyl phthalate), BBP (benzyl-butyl phthalate), and DBP (dibutyl phthalate). Phthalates can be found not only in serum and human urine, but also in milk samples. Tolerable daily intake ranges between 3-30ug/kg/day (Hines *et al*., 2009; Fromme *et al*., 2011; Hannon & Flaws, 2015).

### 3. Atrazine

Atrazine (2-chloro-4-ethylamino-6-isopropylamino-1,3,5-s-triazine), as chlorotriazine, is largely used in agriculture as a herbicide. It has been used to reduce the growth of leaves and weeds in wheat, soy, and sugar cane crops due to the inhibition of photosynthesis (Gianessi, 1998). Its metabolites remain active for long periods of time and, as pesticides, they cause water contamination, including water sources for human consumption (Solomon *et al*., 2013).

### 4. Esters of Polychlorinated and Polybrominated Bisphenols

Polychlorinated bisphenols (PCBs) are chemical substances with a phenolic ring and different degrees of chlorination. They were first manufactured in 1920, and were used in the rubber, resin, adhesives, and paint industries (Soto *et al*., 1995). These chemicals were extensively used around the world and contaminated schools and construction sites. They build up both in the environment and in adipose tissue, and are considered endocrine disruptors affecting the thyroid hormone with estrogenic and anti-androgenic activity. PCBs were banned in 1979 for their persistent pollutant effects. The polybrominated esters of bisphenols were first used as flame retardants and in mattresses and blankets (ATSDR, 2004; 2017). Of all 209 synthesized products categorized as polybrominated aromatic compounds, five esters top the list of toxicity: tetra BDE-47, penta BDE99 -100, -153 and deca BDE-209 or PBDE= Polybrominated diphenyl ethers. (Zota *et al*., 2011; Costa & Giordano, 2007).

### 5. DDT (Dichlorodiphenyltrichloroethane) - DDE (Dichlorodiphenyldichloroethylene) - DDD (Dichlorodiphenyldichloroethane)

These are chemical compounds once widely used as insecticides with a long life and strong lipophilic properties. Evidenced as contaminants to the environment, exposure to these chemicals can lead to several endocrine diseases, although they have been used to control insects that carry malaria (National Toxicology Program, 2011; McGlynn *et al*., 2008; Hardell *et al*., 2004; Safe & Zacharewski, 1997). DDT was banned in 1972 due to its high toxicity levels.

In addition to DDT, other pesticides deserve to be mentioned such as hexachlorocyclohexane, chlordane, and hexachlorobenzene. These products have been closely studied not only for persistently building up in nature but also for being endocrine disrupting chemicals. However, there are new pesticides being launched in the market with shorter mean lives and similar effects, such as 2,4-dichlorophenol, 2,5-dichlorophenol, and 1-naphthol, present in 50% of pregnant women in the Salinas Valley, California, USA.

### 6. Heavy metals or organometallic compounds

Elements such as cadmium, lead, and mercury have been widely used in various scenarios leading to a great number of reproductive anomalies. Cadmium is used in batteries, metallic pigments, and plastics, but exposure to this chemical may cause harmful effects to the placental DNA and fetal umbilical cord, in addition to accumulating in the liver and kidneys. Lead was once extensively used in the paint, oil, and toy industries. Its adverse effects include genomic methylation and a number of different abnormalities in brain development. Mercury was once used in several industrial processes and emissions have been linked to burning charcoal. Human exposure occurs mainly through the intake of contaminated fish from sites such as Minamata Bay, Japan, the Faroe Islands in the Northern Atlantic, and Nunavik in Canada.

### 7. Diethylstilbestrol (DES)

This powerful synthetic non-steroidal estrogen was used in the USA from 1940 to 1975 to prevent miscarriage and/or its complications. Initially, low doses of 5mg/day were administered, but they were progressively increased to 125mg/day or more, and eventually got to a mean dose of 3650-4000mg. Dieckmann *et al*. (1953) proved this treatment was ineffective. Herbst *et al*. (1971) assessed young women and noticed a correlation between the use of DES and the appearance of clear cell vaginal adenocarcinoma. In 1976, the same author (Herbst, 1976) described other abnormalities in the genital tract of young women whose mothers had been treated with DES. Harris & Waring (2012) and Troisi *et al*. (2013) described increased numbers of reproductive system disorders in the male and female children of mothers treated with DES. The disorders included cryptorchidism, uterine abnormalities such as T-shaped uterus, and some types of hormone-dependent cancers.

## Pathological findings

This review lists a number of reproductive abnormalities associated to endocrine disruptors and their different effects:

### 1. Effects of exposure to bisphenols and other toxins on ovarian steroidogenesis

Endocrine glands secrete different hormones that regulate the development, physiologic processes, and homeostasis of all organisms. These hormones interact with various receptors on target cells, according to their affinity, and have dissociation constants ranging between 10-12 and 10-9, associated with their low circulating concentrations. BPA is an endocrine disruptor that binds to estrogen receptors alpha and beta with a binding affinity 1000 to 10000 times lower than that of endogenous estradiol (Kuiper *et al*., 1997; Mlynarcíková *et al.*, 2005). BPA further binds to the gamma and G-protein membrane receptors and to the pregnane X receptor, thus activating ion channels and inducing pro-inflammatory responses of cytokines and chemokines (Chapin *et al*., 2008; Huang & Leung, 2009)

Cholesterol is the substrate needed for enzyme CYP450scc to complete its cleavage and catalyze the conversion from cholesterol to pregnenolone. Pregnenolone is then converted into an androgenic precursor, DHEA (dehydroepiandrosterone), including the intermediary product, 17-Hydroxipregnenolone, involving two enzymes in this conversion: 17 alpha-hydroxylase and 17-20 desmolase. Subsequently, DHEA is converted into androstenedione, which, while in the theca cell compartments, is converted to testosterone, so that both can migrate through the basal lamina of the antral follicle to the granulosa cells. Since there is aromatase CYP450 in the granulosa cells, both androgens are converted into estrone and estradiol (E2) (Two cell theory by Hillier *et al*., 1994).

An experimental study by Peretz *&* Flaws (2013) evaluating female rat ovarian follicles in the antral stage revealed that depending on the dose of BPA administered and the time of action, there was a reduction in the synthesis of estradiol, estrone, testosterone, androstenedione, and DHEA sulfate after 120 hours of exposure to 100µg/ml of BPA. This high level of BPA compromised follicular growth, but no effect was observed following a dose reduction of 1µg/ml (Takayanagi *et al*., 2006). Other experimental studies (Mlynarcíková *et al.*, 2005; Huang & Leung, 2009; Watanabe *et al*., 2012) showed BPA inhibits the mechanism of aromatase CYP450 in the granulosa cells, thus reducing the production of E2.

Only a few studies in humans associated BPA with ovarian follicle synthesis. Mok-Lin *et al.* (2010), Ehrlich *et al*. (2012), and Manikkam *et al*. (2012a) evaluated the relationship between BPA and hormone levels in the granulosa cells of patients submitted to in vitro fertilization, and found a low E2 peak prior to ovum pickup. Lee *et al*. (2014) described found that young people on early puberty exposed to BPA had significant increases in testosterone, estradiol, and pregnenolone levels. On the other hand, Mínguez-Alarcón *et al*. (2015) found no statistical significance between BPA levels found in urine, serum E2 levels, and endometrial thickness measured by ultrasound examination after adjusting the findings for age, body mass index, race, smoking habit, and diagnosis of infertility.

Only a few studies evaluating the harmful effects of exposure to phthalates and the negative effects on steroidogenesis where performed, with insufficient data collection and inadequate statistical methods. On a study called "The Western Australian Pregnancy Cohort Study", Hart *et al*. (2014) described the negative effects of phthalate metabolites on maternal serum SHBG levels, while the association between those same metabolites with the maternal androgen levels was inconsistent. Other animal and in vitro studies described the impact of phthalates on normal steroidogenesis. Xu *et al*. (2010) showed that in rats, aromatase inhibition in the granulosa cells led to decreased E2 levels. Svechnikova *et al*. (2007) and Liu *et al*. (2014) reported decreased progesterone levels in rats exposed to DEHP (diethylhexyl phthalate) and decreased E2 levels in female rats at 20 days of age, but also decreased sexual hormone levels in adult rats even though the results are still controversial.

Claims that exposure to pesticides may change steroidogenesis in women also have their limitations. A previous study by Luderer *et al*. (2013) including 457 Hawaiian participants exposed to heptachlor epoxide, observed a shorter luteal phase and decreased serum levels of progesterone and estradiol metabolites. Atrazine effects in steroidogenesis seem to differ according to age, dose, and experimental model. In vitro studies by Fa *et al*. (2013) showed that Atrazine might alter enzyme expression in steroidogenesis and E2 levels with immature granulosa cells of female rats. In vivo studies with adult animals by Taketa *et al*. (2011), Quignot *et al*. (2012), Tinfo *et al*. (2011), and Buck Louis *et al*. (2014) showed that repeated doses of Atrazine increased enzyme expression in steroidogenesis and sexual hormone levels. An insufficient number of studies have been performed with female patients. Further research is needed to verify whether these pesticides are harmful to steroidogenesis and fecundity. To this day, only Buck Louis *et al*. (2014) with the LIFE Study showed some evidence on the adverse effects of different DEC to female fecundity.

Environmental Toxicants: Su *et al*. (2012) conducted studies in humans showing that high concentrations of dioxins and polychlorinated biphenyl byproducts decreased plasma estradiol levels. Experimental trials with different animals demonstrated that exposure to dioxins adversely affected ovarian steroidogenesis. Further effects included decreases in estradiol production and synthesis in the antral follicles of female rats, and loss of enzyme synthesis (Karman *et al*., 2012a; 2012b). However, there are clear limitations in these trials and further research in humans is required.

### 2. Polycystic ovary syndrome and bisphenols

Polycystic ovary syndrome (PCOS) is an endocrine disorder that includes multiple clinical conditions such as anovulatory cycles, hyperandrogenism, obesity, and regular insulin resistance associated with hypercholesterolemia, dyslipidemia and other metabolic alterations. Today, more than 800 chemical products categorized as endocrine disruptors may strongly affect hormone receptors, act as agonists or antagonists, and lead to anovulation. An evaluation of different phenotypes of patients with PCOS revealed a wide ethnical, geographical, and familial diversity even among twin siblings. Recent studies showed many women exposed to chemical compounds have a genetic susceptibility to developing PCOS and several related metabolic disorders. Time of exposure to these endocrine disruptors is crucial to determine their effect, especially during fetal development, given their potential harmful effects to pregnancy hormones and fetal cellular programming (Palioura & Diamanti-Kandarakis, 2015; Diamanti-Kandarakis *et al*., 2007).

Prenatal androgenization leads to epigenetic changes and future development of PCOS phenotypes (Xu *et al*., 2011). Plastic substances caused DNA methylation in animals, and exposure to biphenyl and phthalates in the F0-generation Zero led to trans-generational changes up to the third generation (F3) (Nilsson *et al*., 2012; Manikkam *et al*., 2012b; 2014). An observational study with female rats given high doses of EDCs such as BPA in the neonatal stage resulted in adult PCOS phenotypes with increased plasma testosterone and estradiol levels, decreased progesterone levels, and development of ovarian cysts (Fernández *et al*., 2010). Fernández *et al*. (2009) also described alterations in the pulsatile secretion of GNRH and LH secretion from the pituitary gland.

Although there is a large number of findings in animal studies, translating their conclusions to humans is rather difficult, once the cystic aspect and the different phenotypes found in animals differ from the findings in humans of PCOS antral follicles. Exposure to testosterone in the beginning of gestation in rhesus monkeys provokes metabolic disorders similar to many women with PCOS. Late exposure to dihydrotestosterone and EDCs causes hyperandrogenism and irregular menstrual cycles, similarly to women with PCOS (Abbott *et al*., 2013).

Coexistence of insulin resistance is found in 50-80% of women with PCOS, leading to decreased insulin sensitivity and hyperglycemia and hyperinsulinemia, followed by hyperandrogenism and chronic anovulation. The mechanisms linked to insulin resistance are still unclear. However, Polyzos *et al*. (2012) suggested the involvement of EDCs in the etiology of insulin resistance. BPA is considered a causal factor of child obesity linked to decreased adiponectin levels, onset of inflammation, and greater risk of developing diabetes type 2 and cardiovascular disorders (Menale *et al*., 2017).

Other mechanisms have been connected to BPA-related hyperglycemia and hyperinsulinemia with direct effect on pancreatic cells, although no alterations on the pancreas islets have been documented (Alonso-Magdalena *et al.*, 2005; 2006).

### 3. Endocrine disrupting chemicals and endometriosis

An enigmatic condition, endometriosis is an estrogen-dependent disease with numerous endocrine disruptors affecting the ectopic endometrial tissue and a wide array of etiological factors. EDCs such as TCDD (2,3,7,8-tetrachlorodibenzo-p-dioxin) and PCBs (dioxin-like polychlorinated biphenyl) may induce the development of peritoneal endometriosis in female Rhesus monkeys, and the magnitude of the effect depends on the level of contamination (Rier *et al*., 2001). Bredhult *et al*. (2007) evidenced by the proliferation of endometrial cells triggered by the angiogenic effect of TCCD’s estrogenization action.

Attention should be paid to The studies with humans performed by Pauwels *et al*. (2001), Eskenazi *et al*. (2002), Fierens *et al*. (2003), Heilier *et al*. (2005), and Simsa *et al*. (2010) deserve attention for the correlations drawn between the effects of dioxin-like products and the genesis of pelvic endometriosis. However, other authors were unable to verify their findings or describe a correlation between PCB and endometriosis (Porpora *et al.*, 2009; Buck Louis *et al*., 2012). 

Phthalates may also have a proliferative effect on endometrial tissue. A prospective case-control study by Kim *et al*. (2011) showed that women with advanced pelvic endometriosis had increased plasma levels of DEHP (di-(2-ethylhexyl) phthalate) and MEHP (mono-(2-ethylhexyl) phthalate) compared to endometriosis-free controls. An additional case-control study found that women with endometriosis had significantly higher concentrations of mono-n-butyl-phthalates in urine than controls (Huang *et al*., 2010). Buck Louis *et al*. (2013) reported levels twice as high of six phthalate metabolites in women with pelvic endometriosis.

Nevertheless, other epidemiological studies failed to validate these findings. Upson *et al*. (2013), in a study including women from the Northeast of the USA, showed an inverse association between the risk of developing endometriosis and levels of MEHP. Itoh *et al*. (2009) confirmed these findings in a study enrolling infertile women, although the authors included only 57 cases of endometriosis and 80 endometriosis-free controls. The mechanism triggering the development of endometriosis by phthalates remains unclear. Only Kim *et al*. (2010) in an in vitro study showed that DEHP (di-(2-ethylhexyl) phthalate) stimulated the stroma of endometrial cells and increased the viability of Ishikawa cells.

### 4. The effects of endocrine disruptors in the ovaries, uterine structure, uterine myomas, and vagina.

In recent years, a number of animal and in vitro studies looked into abnormalities in the development of the ovaries linked to endocrine disruptors. The impact of BPA in the development of human ovaries remains unclear. Previous studies by Rivera *et al.* (2011) and Veiga-Lopez *et al*. (2013) demonstrated that low doses of BPA in sheep might lead to increased ovarian follicles with multiple oocytes and altered ovarian steroidogenesis. Hunt *et al*. (2012) described the impact of BPA in the fetal development of female monkey ovaries affecting early meiosis, causing synaptic alterations, and interfering with the recombination between homologous chromosomes.

Insufficient data is available on the impact of phthalates, pesticides, and other environmental toxicants in human prenatal ovarian development. Studies on human postnatal ovarian development are also limited. Sheep and rats exposed to BPA during pregnancy had ovarian anomalies. Low doses of BPA decreased the number of follicles and increased follicular atresia in female rats. On the other hand, high doses of BPA might lead to increased follicular cystification, corpus luteum depletion, and decreased antral follicle counts (Rodríguez *et al*., 2010; Delclos *et al*., 2014). According to Chen *et al.* (2012), human ovaries exposed after birth to phthalates such as benzyl butyl phthalate have greater chances of developing granulosa cell apoptosis.

Numerous pesticides such as endosulfan, malathion chlorpyriphos, and cypermethrin cause postnatal ovary anomalies, inducing decreased follicle counts and increased follicular atresia as described by Koç *et al*. (2009) and Nandi *et al*. (2011). While looking into another noteworthy environmental toxicant, Petro *et al*. (2012) linked increased levels of chlorinated bisphenols in humans and in ovarian follicular liquid to decreased fertilization rates and poor conditions for oocyte development.

The uterus is a muscle organ consisting of two main elements: the body, which includes the endometrium, and the caudal end, or cervix, both exposed to significant hormonal influence from their early stages of development. After puberty, the uterus has periodical cycles of hormonal variation and greatly expands during pregnancy, while after menopause the uterus involutes and decreases in size.

The prospective effects of BPA and pesticides in uterine structure and function remain unknown, but abnormalities have been reported in animal studies. Exposure to BPA in the gestational and neonatal periods causes the development of endometrial glands and stroma, as seen in the adipose tissue next to the genital tract of type Balb-c-adult female rats (Signorile *et al.*, 2010; 2012). A single Australian study found that human exposure to mono (carboxy-isooctyl) phthalate changed the uterine volume (Hart *et al*., 2014). As previously demonstrated (Dieckmann *et al.*, 1953; Herbst *et al.*, 1971; Herbst, 1976; Harris & Waring, 2012; Troisi *et al.*, 2013), diethylstilbestrol caused a great number of uterine abnormalities in the exposed daughters of pregnant women treated to prevent miscarriage during gestation. Along the same lines, other recent studies showed that exposure to DES induced endometrial hyperplasia/dysplasia and increased the chances of endometrial adenocarcinoma and uterine anomalies in female rats and hamsters (Alwis *et al*., 2011; Yoshida *et al*., 2011).

Other environmental toxicants have been tested in animals and in humans. Su *et al*. (2012) found that dioxin and polychlorinated aromatic byproducts of biphenyls caused anomalies in the uterine structure, function, and fundus of 33 young girls. Uterine myomas or fibromyomas are mostly benign tumors, affecting approximately 70-80% of the female population throughout their lives. The growth of nodular tumors and multiple myomas is hormone-dependent and connected to the estradiol and progesterone receptors in the myometrium. The estradiol produced in the granulosa cells of the ovarian follicles regulates the endometrium and myometrium cells by activating their alpha and beta cellular receptors. The binding affinity between estradiol and its receptor can trigger a number of events, mostly in the cell nucleus, by recruiting important proteins to cellular reproduction.

Following ovulation, the corpus luteum produces progesterone, an essential hormone to female reproduction, binding to the A and B progesterone receptors. These receptors promote and regulate the expression of several genes, leading to different cellular responses. No significant correlation has been found between BPA activity and its capacity to promote the development of fibromas. Two Chinese case-control studies by Shen *et al*. (2013) and Zhou *et al*. (2013) showed an association between higher levels of BPA, nonylphenol and octylphenol in women with uterine myomas.

Other authors found positive correlations between disease and phthalates. In 2010, the NHANES study showed that mono-benzyl phthalate increased the risk of myoma in 1227 women. However, other phthalates such as MEHP (mono-(2-ethylhexyl) phthalate), MEHHP (mono-(2-ethyl-5-hydrohexyl) phthalate), and EOP (mono- (2-ethyl-oxohexyl) phthalate) were inversely correlated (Weuve *et al*., 2010) with the onset of disease.

Meanwhile, few studies reported that prenatal exposure to DES (diethylstilbestrol) increased the number of fibromas. The NURSES study included 11831 and followed them for over 20 years. DES exposure during gestation increased the risk of fibroma by 13%, while DES exposure during the first trimester of pregnancy increased the risk of fibroma by 21% in comparison to non-exposed women (Baird & Newbold, 2005; Mahalingaiah *et al*., 2014). Another NIEHS Uterine Fibroid Study completed in Washington-DC including 1364 exposed women aged 35-49 reported an odds ratio of 2.4 for Caucasian women (Baird & Newbold, 2005). Subsequently, the NIEHS Sister Study evaluated a group of 3534 African-American women and showed increased risk of developing fibromas after exposure to DES in women with maternal and gestational diabetes and women pregnant with monozygotic twins, with respective odds ratios of 2.02, 1.54, and 1.94 according to D'Aloisio *et al*. (2012). This study included 19972 Caucasian women and documented five significant risk factors: prenatal exposure to DES; gestational diabetes; getting pregnant while having a history of diabetes; use of soy protein-based formula; and advanced maternal age. All these factors represented an increase of more than 20% in the risk of having fibromas.

As described in 1953, chemical disruptors such as DES (diethylstilbestrol) induce the development of neoplasms such as vaginal adenosis and clear cell adenocarcinoma on the vaginal walls (Dieckmann *et al*., 1953; Herbst *et al*., 1971; Herbst, 1976; Harris & Waring, 2012; Troisi *et al*., 2013). DES (Diethylstilbestrol) inhibits the vaginal stroma causing a persistent down-regulation of the transcription factors (Laronda *et al*., 2013; Katoh *et al*., 2013; Nakamura *et al*., 2012a; 2012b).

### 5. Effects of exposure to endocrine disrupting chemicals on the pituitary gland, menstrual cycles, and fertility

Only a handful of human studies have looked into the correlation between bisphenols, phthalates, and pesticides and harmful effects on the anterior pituitary gland compartment. On the other hand, Xi *et al*. (2011) and Brannick *et al*. (2012) showed that prenatal and immediate postnatal exposure to BPA stimulated the hypothalamic-pituitary axis and increased the number and replication of pituitary gonadotropins in female rats. A recent study by Souter *et al.* (2013) found no correlation between exposure to BPA and FSH levels in women on the third day of IVF cycles. Miao *et al*. (2015) described a positive correlation between exposure to BPA and urinary levels of prolactin, although a negative correlation was established with FSH levels.

Animal studies have occasionally described harmful effects of other toxicants such as DES and dioxins (Yoshida *et al*., 2011; Ishikawa *et al*., 2014) as having rather controversial effects on the pituitary gland and human gonadotropins. Insufficient human studies were made in the last five years with regard to the effects of toxicants on fertility and menstrual cycles. According to Buck Louis *et al*. (2011), organochlorine pollutants led to an increase of three additional days on the interval between the periods of women willing to conceive. Few studies demonstrated abnormalities in the menstrual cycles due to exposure to phthalates. Exposure to BPA induced abnormalities in the estrous cycle of rats according to Fernández *et al*. (2009), and Nah *et al*. (2011). Vélez *et al*. (2015), in the MIREC Study the Maternal-Infant Research on Environmental Chemicals, included 2001 women in the first trimester of pregnancy exposed to bisphenols and phthalates and the time of conception. Higher concentrations of Triclosan (>72ng/ml), a bactericide with phenolic compounds, decreased fertility, but no correlation was found with bisphenols and phthalates.

In Brazil, for many years several authors have shown their concern and have looked into the effects of environmental EDCs on the population and as a factor in occupational health (Branco, 1984; Nogueira *et al*., 1987; Della Rosa & Gomes, 1988; Assunção & Pesquero, 1999; Sanseverino *et al*., 2001). Studies by Peres *et al*. (2001), Lara *et al*. (2011), and Cremonese *et al*. (2012) also evaluated EDC potential harmful effects in the field of reproductive health.

## CONCLUSIONS

Growing outspread exposure to a number of different chemicals has caused multiple abnormalities in the reproductive system of women and different animal species. Tens of millions of these chemicals are available in the global market, and even when administered in minimal doses, they have been described as endocrine disrupting chemicals or potential conditioners. Exposure to EDCs is extremely toxic and harmful to the reproductive system. Despite the occasional absence of a match between experimental and epidemiological data, it is clear that EDCs can produce adverse effects on the genital tract. The reproductive effects may be dose-dependent and associated to extended exposure and the level of activity of the compound at hand. Unfortunately, a significant number of these compounds are already a part of the environmental chain, making it difficult to assess their potential harmful effects.

Di Renzo *et al.* (2015) in the FIGO (International Federation of Gynecology and Obstetrics) drafted the following guidelines that must be observed by gynecologists, obstetricians, nurses, and other healthcare workers in this field:

- Extensive guidance and education to ensure preventive measures are in place with respect to exposure to toxicants;
- Appropriate measures to ensure healthy food is provided in the entire food chain; make sure pesticides are banned from farms so as not to contaminate vegetables, fresh fruits, or whole wheat grains; reduce the intake of animal fat or fish contaminated by heavy metals;
- Educational and participatory guidance involving the entire society to ensure they are aware of the risks related to the intake of these toxicants.

Further prospective studies are needed to better elucidate the possible abnormalities affecting women by millions of EDCs.
